# Shaping Liquid Droplets on an Active Air–Ferrofluid Interface

**DOI:** 10.1021/acs.langmuir.3c00298

**Published:** 2023-05-24

**Authors:** P. A. Diluka Harischandra, Teemu Välisalmi, Zoran M. Cenev, Markus B. Linder, Quan Zhou

**Affiliations:** † Department of Electrical Engineering and Automation, School of Electrical Engineering, Aalto University, 02150 Espoo, Finland; ‡ Department of Bioproducts and Biosystems, School of Chemical Engineering, Aalto University, FI-00076 Espoo, Finland; § Department of Applied Physics, School of Science, Aalto University, 02150 Espoo, Finland

## Abstract

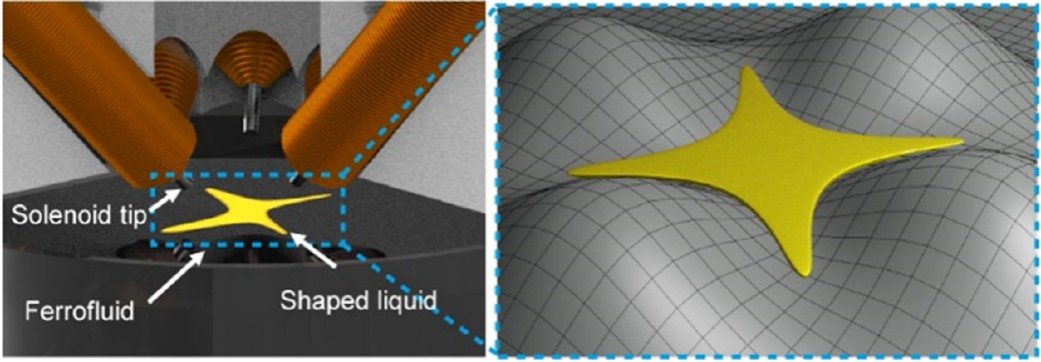

An air–liquid interface is important in many biological and industrial applications, where the manipulation of liquids on the air–liquid interface can have a significant impact. However, current manipulation techniques on the interface are mostly limited to transportation and trapping. Here, we report a magnetic liquid shaping method that can squeeze, rotate, and shape nonmagnetic liquids on an air–ferrofluid interface with programmable deformation. We can control the aspect ratio of the ellipse and generate repeatable quasi-static shapes of a hexadecane oil droplet. We can rotate droplets and stir liquids into spiral-like structures. We can also shape phase-changing liquids and fabricate shape-programmed thin films at the air–ferrofluid interface. The proposed method may potentially open up new possibilities for film fabrication, tissue engineering, and biological experiments that can be carried out at an air–liquid interface.

## Introduction

An air–liquid interface is important in studies of respiratory infectious disease,^1,2^ toxicology,^3^ and biofilms,^4^ as well as industrial applications such as the fabrication of lenses^5^ and formation of monolayers on surfaces.^6^ The advancement of these applications often depends on the manipulation capabilities of liquid droplets at the air–liquid interface. Several studies have proposed techniques to transport droplets at the air–liquid interface. Oil droplets can be transported on the air–magnetic liquid interface by deforming the interface using permanent magnets.^7^ Oil droplets can be also moved by deforming the air–liquid interface using an air bubble.^8^ The motion of aqueous droplets over spikes of oil-based ferrofluid droplets has also been studied using magnetic fields.^9^ Furthermore, liquid droplets and nonmagnetic colloidal particles can be transported and trapped on ferrofluid-infused microstructured surfaces with reconfigurable multiscale topographies.^10^ However, those manipulation capabilities are limited to transportation and trapping, and more advanced manipulation capabilities such as the shaping of droplets are largely not studied besides the patterning of a group of droplets.^11^

Shaping of droplets has previously been studied in other environments. Most of the works directly manipulate the liquids using energy fields such as airflow, acoustic fields, magnetic fields, and electric fields in fluidic media such as air and liquid. Airflow^12^ or acoustic fields^13^ can levitate liquid droplets and produce star-shaped oscillations. Oil droplets in an oil–water emulsion can be deformed into hexagonal platelets, triangular platelets, or long asperities.^14^ Liquid crystal droplets in an aqueous solution can be shaped into elongated filaments at a certain cooling rate.^15^ Spontaneous patterns of nonequilibrium fluidic lattices and droplets of polygonal or toroidal shapes may occur in two oil mixtures under electric fields.^16^ A ferrofluid droplet can transition and back-transition from the droplet form into spikes as a response to an approaching magnet.^17^ A ferrofluid droplet in ethanol may be deformed into an elliptical shape under a magnetic field.^18^ Aqueous droplets immersed under a thick layer of ferrofluid can be deformed, attracted, and coalesced using magnetic fields.^19^ Besides, liquid shaping can also be carried out on a solid surface. A water droplet residing on a superhydrophobic surface can be elongated using photothermally induced pyroelectric effects.^20^ Ring-shaped or C-shaped ferrofluid droplets have also been employed to transport hydrogel balls using ring magnets.^21^ Liquid metal droplets can also be moved or stretched to a cross-shaped pattern using magnetic fields when mixed with iron nanoparticles.^22^ Droplets can be also transported on magnetic-sensitive surfaces by altering the wettability of the surface using a ring-shaped magnet.^23^ Those works have achieved impressive results; however, they have little applicability to the shaping of liquid on the air–liquid interface.

**Figure 1:**
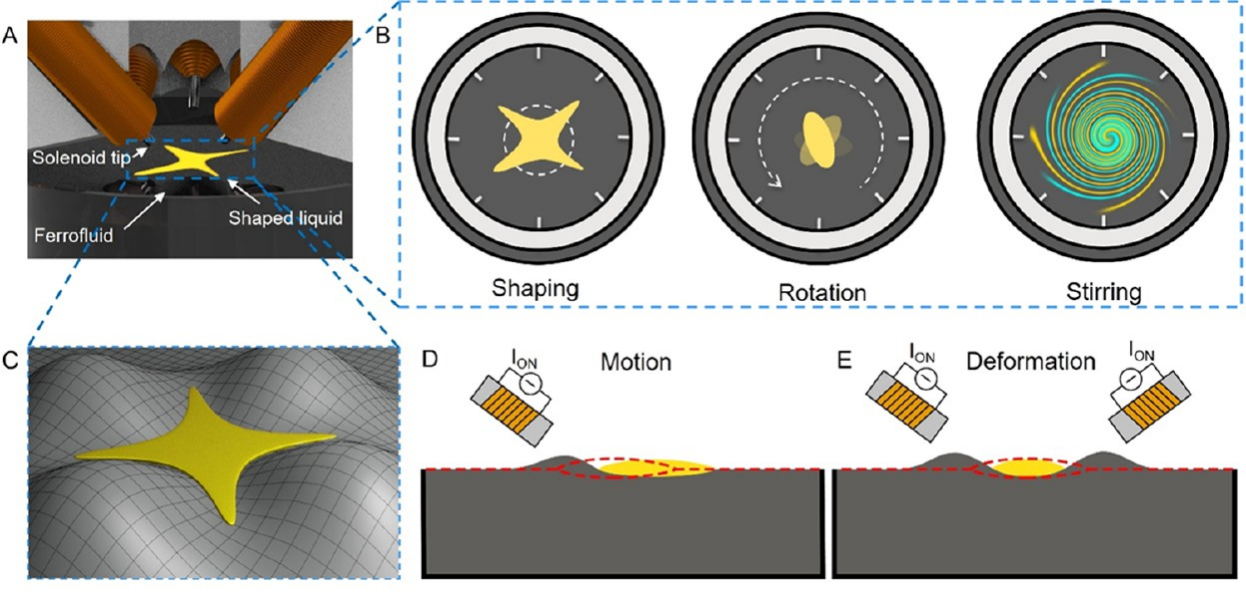
Concept of liquid shaping on the air–ferrofluid interface. (A) An assembly of eight solenoids above an air–ferrofluid interface with a liquid droplet, where the solenoids can deform the interface to shape the liquid. The experimental setup was adapted from our previous work.^24^ (B) Shaping, rotating, and stirring a liquid droplet. (C) 3D illustration of the deformation of the air–ferrofluid interface leading to the shaping of the liquid droplet. Illustration of (D) the motion and (E) the deformation of a liquid droplet on different deformed air–ferrofluid interfaces (not to scale). The red dashed lines show the initial state of the oil droplet.

Here, we report a multicoil magnetic shaping method that can statically and dynamically squeeze, rotate, and shape nonmagnetic liquids on a programmable deformable air–ferrofluid interface, as illustrated in Figure 1. We can control the droplet morphology on demand in good repeatability by programming the external magnetic fields, where the shapes of the droplet can be either convex or concave. We can squeeze a hexadecane droplet at different aspect ratios and rotate it. Using rotational excitation, we can also stir oil droplets into spiral-like structures. Additionally, we can shape phase-changing liquid that solidifies at the air–ferrofluid interface, resulting in polystyrene films in several programmable shapes.

## Results and Discussion

### Working Mechanism

To shape liquid on the air–ferrofluidic liquid interface, we employ eight independently controlled solenoids arranged around the manipulation area, illustrated in Figure 1A. We can program the deformation of the air–ferrofluid interface using external magnetic fields generated from the solenoids. The curvatures created at the air–ferrofluid interface are used to manipulate liquid droplets on the interface, to achieve shaping, rotation, or stirring, as illustrated in Figure 1B. Figure 1C shows the concept of shaping a droplet on the air–ferrofluid interface, where an X-shaped droplet is formed by generating four bumps on the interface. Details of the experimental setup are explained in the Experimental Section.

The working principle of the liquid shaping system is illustrated in Figure 1D. The black and red dashed lines represent the states of the droplet before and after deformation created by the solenoid, respectively. The motion of the droplet on the air–ferrofluidic interface is a combined effect of the gravitational field, magnetic interaction, and interfacial tension, which can be described as an energy minimization problem with the total energy similar to the case of the motion of solid particles at the air–magnetic liquid interface we developed previously.^25^ Hence, the total energy of an oil droplet at the air–ferrofluid interface under nonuniform magnetic field becomes
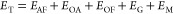
1where *E*_AF_, *E*_OA_, and *E*_OF_ are the interfacial energies of an oil droplet at a flat air–ferrofluid interface, an oil–air interface, and an oil–ferrofluid interface, respectively; *E*_G_ is the gravitational potential energy; and *E*_M_ is the magnetic energy. The gravitation potential energy *E*_G_ is a function of the effective mass of the droplet *m*_eff_ = ϱ_O_*V*_O_ – ϱ_F_*V*_imm_, the gravitation acceleration, and the interface deformation of the ferrofluid caused by the magnetic field. ϱ_O_ and *V*_O_ are the density and volume of the oil droplet, respectively, ϱ_F_ is the density of the ferrofluid, and *V*_imm_ is the displaced volume of the ferrofluid by the oil.

The behavior of oil droplets such as hexadecane is similar to high-density particles at the air–ferrofluid interface where the droplets get repelled from the bump created at the air–ferrofluid interface. This could be attributed to the positive effective mass arising from the similar densities of both the hexadecane droplet (0.77 gcm^–3^) and the ferrofluid (0.99 gcm^–3^). The confinement of a droplet between two magnetic field-induced bumps at the air–ferrofluid interface is illustrated in Figure 1E. By actuating a carefully chosen combination of solenoids, we can also create several bump-like convex deformations, which confine the droplets in the directions of the bumps and expand the droplet in the directions of the valleys as shown in Figure 1C.

### Quasi-Static Squeezing of a Hexadecane Droplet

**Figure 2:**
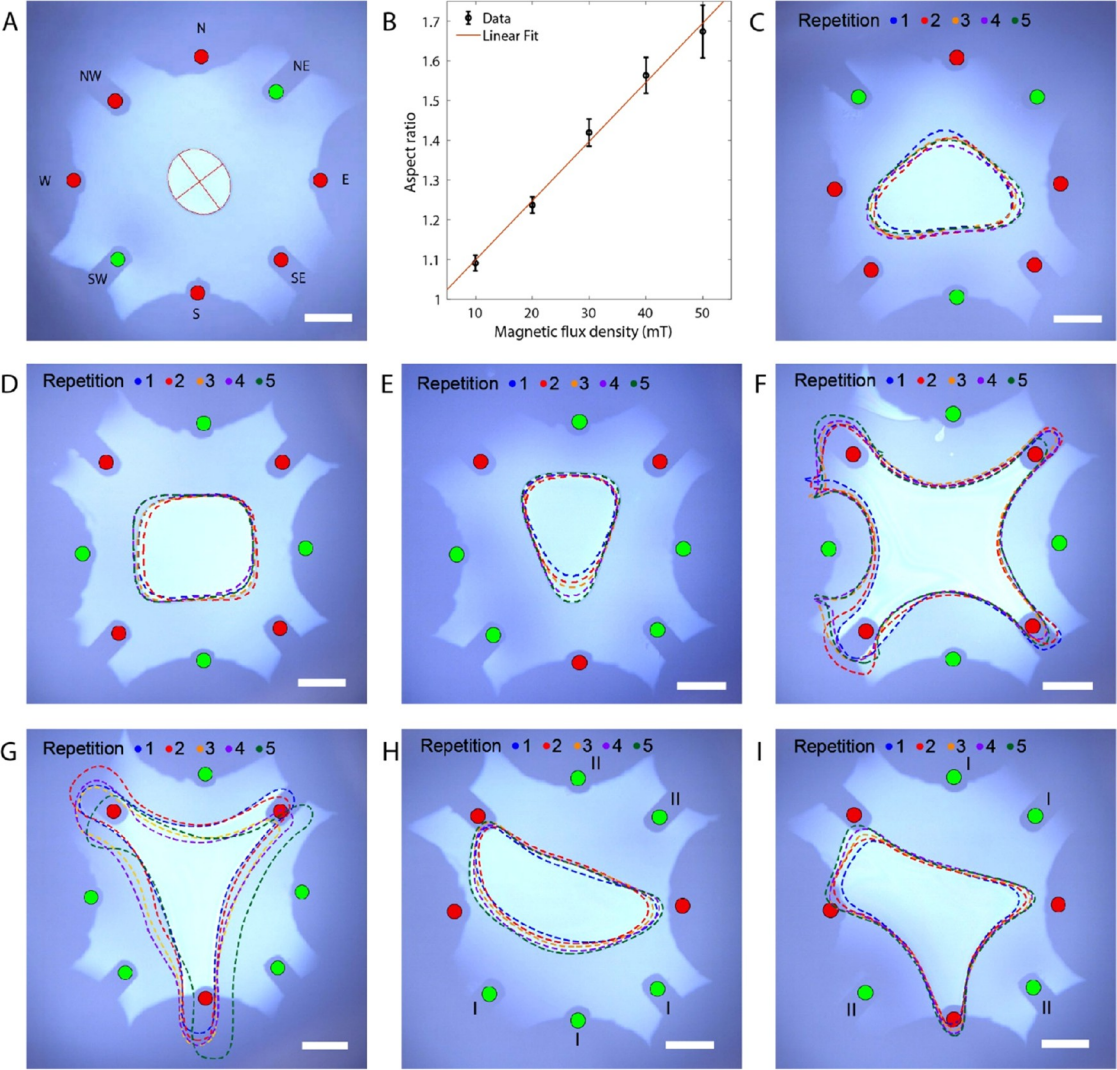
Shaping a hexadecane droplet on an air–ferrofluid interface. (A) Shape response of a hexadecane droplet when two opposite solenoids are actuated. (B) Aspect ratio of the fitted ellipse when the solenoids are actuated with different magnetic flux densities at the solenoid tip. (C–E) Shape response when several solenoids are actuated simultaneously. (F–G) Shape response when several solenoids are actuated after 15 min from the initial placement of the droplet at the air–ferrofluid interface. (H, I) Shape response for sequential actuation of the solenoids. The sequence order is shown by the number next to the actuated solenoid indicator. Scale bars are 2 mm. Active solenoids are marked with green circles and inactive ones with red circles.

We actuated different combinations of multiple solenoids to investigate the variety and repeatability of resultant shapes using a hexadecane droplet. Figure 2A shows the deformation of an initially circular hexadecane droplet (∼0.2 μL) after five seconds of actuation using two opposite solenoids at 28 mT, where the actuated solenoids are marked with green dots and the different solenoids are denoted by cardinal and intercardinal directions. The volume of 0.2 μL was empirically selected to prevent the droplet from spreading past the workspace boundaries for the duration of the repeatability studies. We analyzed the aspect ratio of the resultant ellipse shapes for five different magnetic flux densities (10, 20, 30, 40, and 50 mT) measured at ∼500 μm from the solenoid tips, where each experiment was repeated five times. The aspect ratio of the fitted ellipse was 1.09 ± 0.02 at 10 mT and linearly increased to 1.67 ± 0.07 at 50 mT, as shown in Figure 2B, with a linear relationship of *AR* = 0.015*B* + 0.95 (also shown in Movie S1). The increase in the magnetic flux density also led to an increase in the standard deviation of the aspect ratio, which can be attributed to the increased nonlinearities due to larger deformations of the air–ferrofluid interface at higher magnetic flux density.

We can also produce nonequilibrium shapes when multiple solenoids are actuated simultaneously. Ideally, eight solenoids produce  different actuation combinations, where *k* denotes the number of simultaneous actuations. The circular arrangement of the solenoids reduces the number of combinations to 29 unique actuation combinations. However, a droplet will be pushed out of the workspace if most of the actuated solenoids are on one side of the workspace. Therefore, we demonstrate the combinations that can keep the droplet near the center of the workspace.

The formation of triangular and square shapes is shown in Figure 2C–E. Before each shaping trial, the droplet is centered and all of the solenoids are demagnetized by applying a demagnetization waveform (see Supporting Figure S4). Then, the desired combination of solenoids is actuated with a ramp-up signal to 25 mT in one second and is held at that level for four seconds, where the process is repeated five times for each shape. The resultant shapes in adjacent trials show good repeatability with areas of 17.18 ± 0.49, 19.37 ± 0.61, and 13.42 ± 1.34 mm^2^ for the convex shapes shown in Figure 2C–E, respectively. An increase in area with each repetition is observed in the repetitions due to the spreading of the droplet at the air–ferrofluid interface. The spreading behavior of the oil droplet could be described with the spreading coefficient *S* = γ_AF_ – γ_OA_ – γ_OF_, where γ_AF_ and γ_OA_ represent the surface tensions of the ferrofluid and oil droplet and γ_OF_ represents the interfacial tension of the oil droplet and the ferrofluid.^26^ The spreading coefficient for a hexadecane droplet at the air–ferrofluid interface was 1.64 (see Supporting Table S2, Technical Note S1). The positive spreading coefficient indicates that the droplet will spread and is consistent with our observations. This spreading phenomenon of the droplet has a major contribution to the resultant shape since it changes the relative size of the droplet and the deformed interface. For instance, the shapes can transform from convex to concave approximately 15 min after the initial placement of the droplet at the air–ferrofluid interface. Therefore, by actuating the same solenoid combinations used for convex shapes shown in Figure 2D,E, we observed concave shapes shown in Figure 2F,G after ∼15 min due to the spreading phenomenon of the droplet (Movie S2 parts I, II). The contour shapes at the regions obscured by the manipulator were approximated. During the recentering of a droplet, a droplet separation can occur at regions directly under the solenoid tips, as shown in Figure 2F near the north solenoid.

Multiple solenoids on both sides of the workspace can also be actuated sequentially to form diverse shapes of the droplet. The opposite motions of the droplet generated by the two sides sequentially will cancel out and maintain the droplet in the workspace. In Figure 2H, we iteratively actuated S, SE, and SW solenoids first and then the N and NE solenoids second (also shown in Movie S2, part III, sequence A). In Figure 2I, we show the resultant shape when the NE and E solenoids are actuated first and then SW and SE solenoids are actuated second (Movie S2, part III, sequence B). The resultant shapes are similar in the repeated experiments.

The experimental results show that a variety of shapes including ellipses, half-moons, triangles, and quadrilaterals can be created. Additionally, both concave and convex shapes can be generated for triangles and quadrilaterals. All corners of the shapes are roundish with a diameter of at least 0.3 mm due to the surface tension. The shapes can be reasonably recreated between repetitions, where the best case can vary by 2.84% in the area.

### Rotation of a Hexadecane Droplet

**Figure 3:**
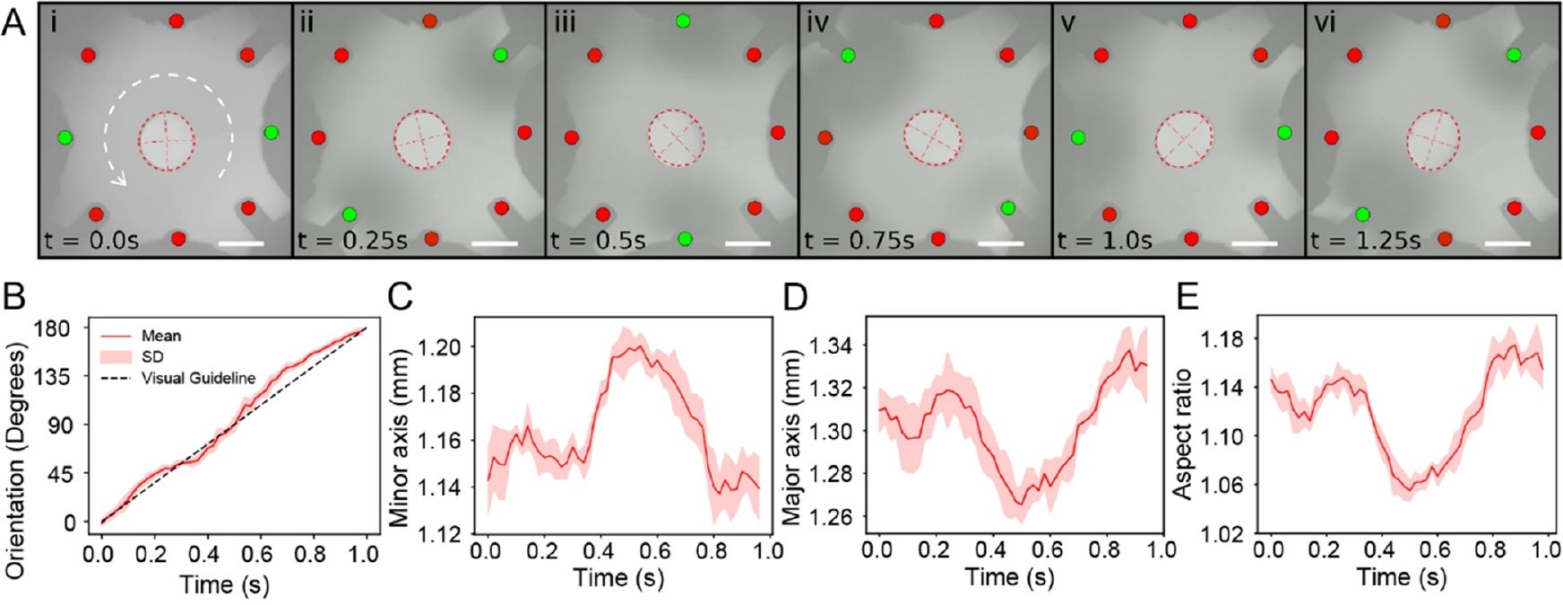
Dynamic response of hexadecane droplet when the solenoids are actuated with sinusoidal waves. (A) Timelapse of the droplet rotation. (B) Angle response of the rotating droplet. (C) Minor axis length of the droplet. (D) Major axis length of the droplet. (E) Aspect ratio of the droplet. The mean and standard deviation are represented by the red line and the shaded area, respectively. The black dashed line shows the visual guideline. Scale bars are 2 mm. Active solenoids are marked with green circles and inactive ones with red circles.

We also rotated a hexadecane droplet on the air–ferrofluidic interface by creating a rotating magnetic field with sequential actuation of the opposite solenoid pairs, which are (NE, SW), (W, E), (NW, SW), and (N, S). Liquid droplets have been previously rotated using magnetic fields^18,27^ or electric fields,^28^ which however require the liquid to possess certain electric and magnetic properties. To squeeze the hexadecane oil droplet, two opposite solenoids are actuated with sinusoids of the same phase and adjacent solenoids are actuated with sinusoids of 90° phase difference with a peak magnitude of 39 mT at the solenoid tips (see Supporting Figure S5). Only the positive half-cycles of the sinusoids were used, while the negative half-cycle is set to zero. Therefore, two cycles of the sinusoid-like signals are needed for each rotation of the droplet. Figure 3A shows the timelapse of how an initially circular hexadecane droplet deforms into an elliptical shape and starts to rotate when the solenoids are actuated with a 0.5 RPS spinning rate (Movie S3). The droplet can rotate with full cycles until 0.5 RPS. Beyond that, the droplet is unable to follow the complete cycle. In Figure 3B, we show the orientation of the droplet rotating from 0 to 180° for five cycles at a spinning frequency of 0.5 RPS. The angular velocity of the droplet is generally linear (*R*^2^ = 0.97) and repeatable (see Figures 3B, S5D–G). The minor deviations could be attributed to the oscillation of the liquid surface and the difference in the remanence magnetic fields of each solenoid. The resultant rotating droplet has a major axis of 1.30 ± 0.02 mm, a minor axis of 1.17 ± 0.02 mm, and an aspect ratio of 1.12 ± 0.04 (Figure 3C,D). Both the major axis and minor axis lengths were increasing with each cycle due to the spreading of the droplet at the air–ferrofluid interface.

### Stirring of Liquid Dispersed at the Air–Ferrofluid Interface

**Figure 4:**
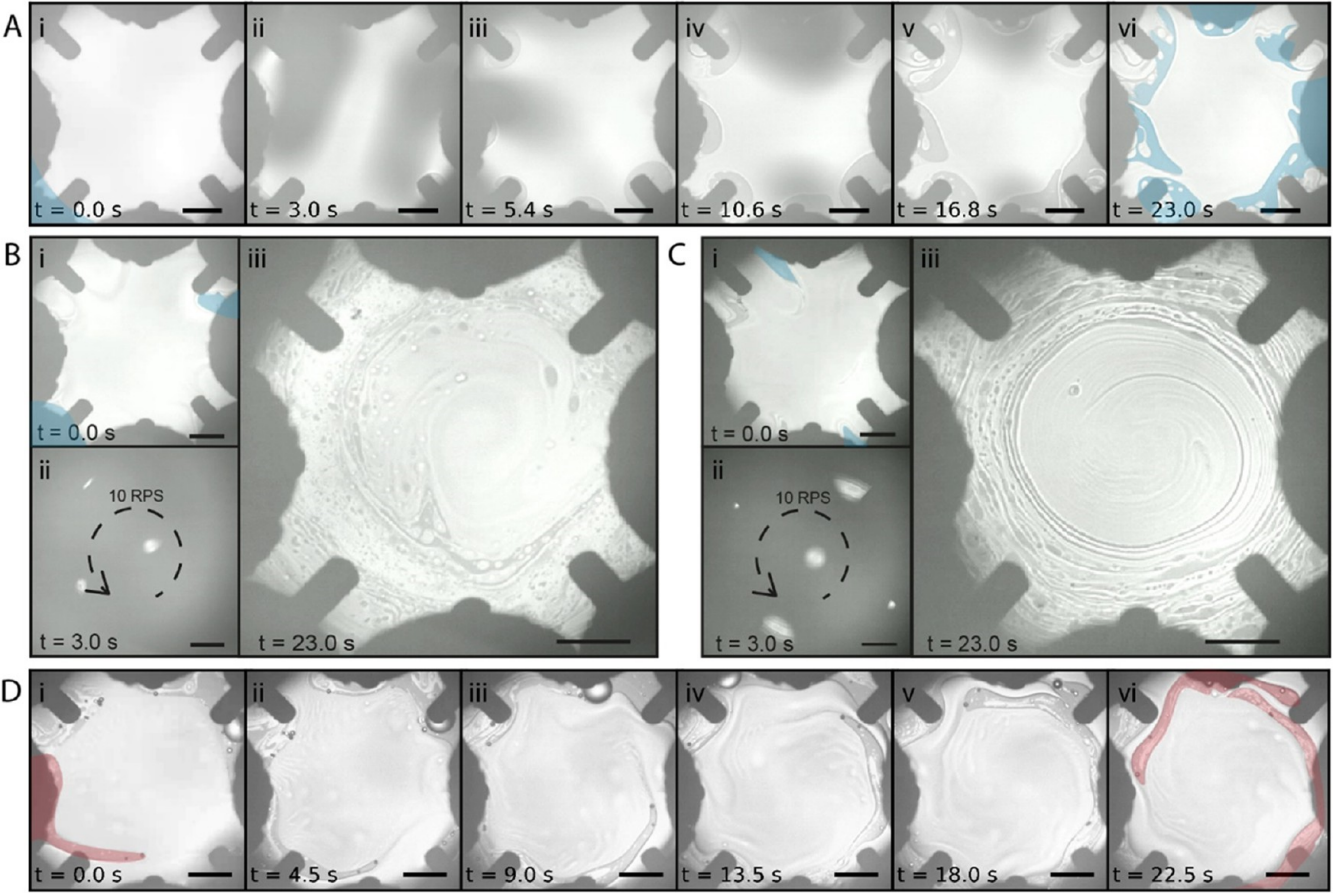
Stirring of oils dispersed at the air–ferrofluid interface. (A) 5 RPS stirring of 10 cSt silicon oil at the air–ferrofluid interface. The shaded blue area shows the ferrofluid surface. (B) 10 RPS stirring of 10 cSt silicon oil. (C) 10 RPS stirring of 1000 cSt silicon oil. (D) 100 RPS stirring of hexadecane and 10 cSt silicon Oil. The shaded red area shows the motion path of the hexadecane droplets. The brightness and contrast of the images have been adjusted to improve readability. Scale bars are 2 mm.

Besides rotating droplets, we also investigated the stirring of oils dispersed on the air–ferrofluid interface. Stirring is crucial for improving the liquid mixing efficiency.^29,30^ We employed silicon oil for its relatively low surface tension (γ_Si-oil_ = 20.1 mN m^–1^) and a high spreading coefficient (*S*_Si-oil_ = 16.71) at the air–ferrofluid interface compared to the one of hexadecane (γ_C_16_H_34__ = 27.39 mN m^–1^) and *S*_C_16_H_34__ = 1.64. By applying a rotational magnetic field as in the previous section, we created spiral-like motion on the dispersed oil droplet. Figure 4A shows the timelapse of stirring a silicon oil droplet (∼0.2 μL) with a viscosity of 10 cSt at a spinning frequency of 5 RPS (Movie S4 part I). At spinning frequencies of 5 RPS or lower, the droplet barely rotated or remained still. The silicon oil droplet broke into smaller droplets in the vicinity of the solenoid tip, which we attribute to the higher deformation of the ferrofluid near the solenoid tip. However, when the spinning frequency is greater than 5 RPS, we observed spiral-like motions of the silicon oil. Figure 4B,C shows the stirring response when the solenoids were actuated with 10 RPS spinning frequency for 20 s for silicon oil with 10 and 1000 cSt viscosities, respectively (Movie S4 part II). The deformation of the ferrofluid was the greatest at 10 RPS and distorted the video due to extensive scattering of the light. We denote this frequency as the critical frequency of the system.

We also stirred two immiscible liquid mixtures, where we placed droplets of silicon oil (∼0.2 μL) with a viscosity of 10 cSt and hexadecane (∼0.4 μL) on the same ferrofluid surface. We used sinusoids of 200 Hz frequency or spinning frequency of 100 RPS to minimize the scattering of light and to observe the stirring process. Figure 4D shows the stirring of the two oils (Movie S4 part III). The silicon oil is dispersed throughout the workspace similar to the previous experiments. On the contrary, hexadecane forms droplets, visible as dark dots at the air–ferrofluid interface. The hexadecane droplets at the edge of the workspace form a snake-like motion occurred from the combined rotational motion of the ferrofluid and the pushing motion toward the center of the workspace caused by the deformation of the air–liquid interface when the hexadecane droplet passes by each solenoid.

We attribute these rate-dependent stirring results to the relative dominance of gravitational force and viscosity at different rates. At a slower spinning rate, the system is similar to quasi-static; therefore, gravity dominates and the oil droplet slides down the bump similar to the shaping experiments. At a high spinning rate of 10 RPS or greater, the viscosity dominates, and the ferrofluid pulled by the solenoid may not be fully at rest before the next solenoid is activated, so the ferrofluid near the previous solenoid may be pulled toward the next solenoid, leading to a net motion of the ferrofluid. Consequently, spiral-like structures may form as a result of oil being dragged with the ferrofluid. The resulting formation of the spiral also depends on the viscosity of the oil. At a low viscosity, 10 cSt, the surface tension dominates, so the spiral will be composed of droplets. At a high viscosity of 1000 cSt, the viscosity dominates over surface tension, so the droplet is more in the form of ribbons. This can be explained using Laplace number La = ϱγ*l*/η^2^, where ϱ, γ, *l*, and η denote the density, surface tension, characteristic length (droplet volume radius), and viscosity of the droplet, respectively. For hexadecane, and two silicon oils, viscosities 10 and 1000 cSt, the Laplace numbers are 603.22, 65.20, and 0.0061, respectively. The experiments demonstrate that, when the surface tension is dominating, i.e., a high Laplace number, droplets can be shaped or rotated, whereas when the viscosity is dominating, i.e., a low Laplace number, droplets can be stirred, where the stirred liquid can still form droplet for a Laplace number of 65.2 but more ribbon-like for a Laplace number of 0.0061.

### Formation of Polystyrene Films on the Air–Ferrofluid Interface

**Figure 5:**
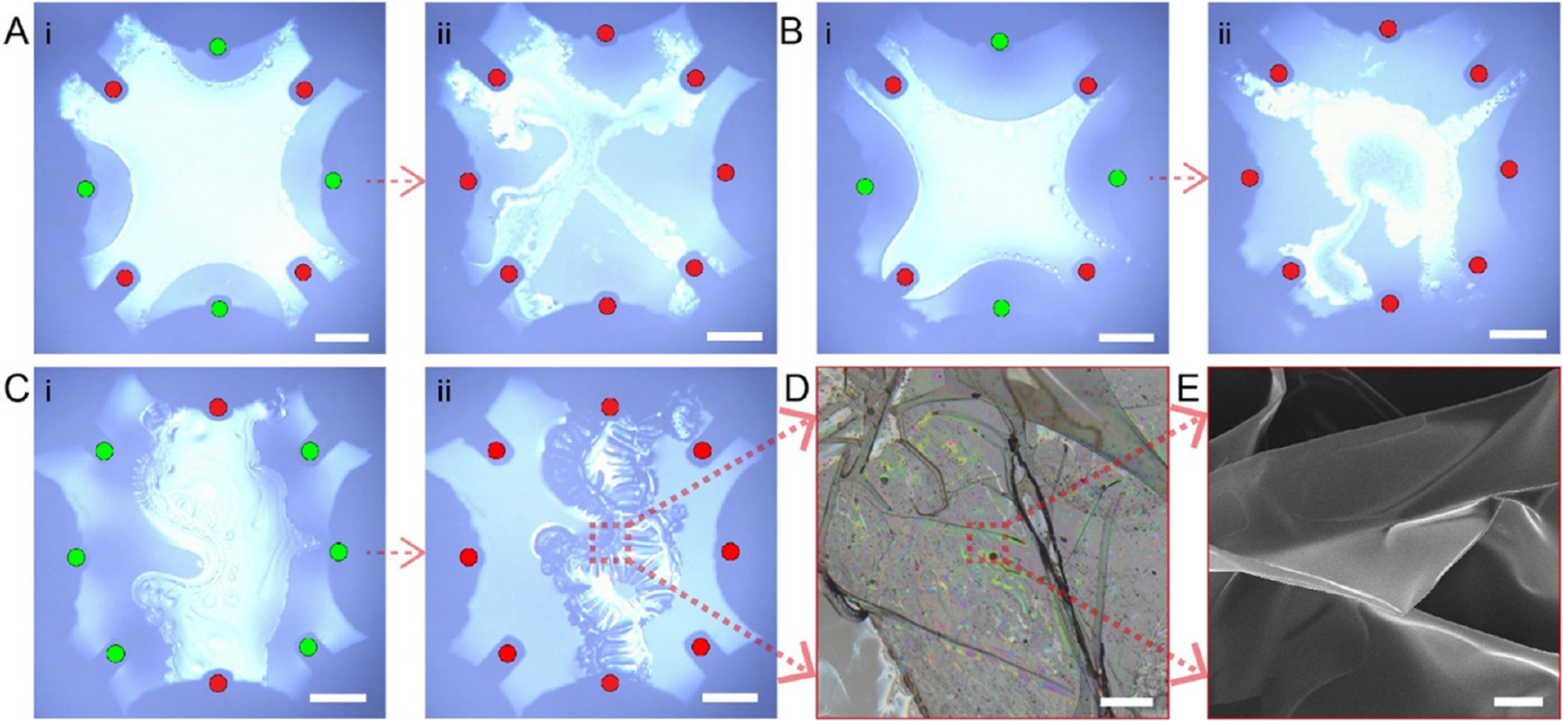
Shaping and formation of films at the air–ferrofluid interface. (A, B) Formation of a film using 1.7 mg/mL polystyrene toluene solution. (C) Formation of a film using 50 mg/mL polystyrene/toluene solution. Active solenoids are marked with green circles and inactive ones with red circles. (D) Formed polystyrene film under the microscope. (E) Scanning electron microscopy image of the formed polystyrene film. Scale bars are 2 mm for panels (A–C), 200 μm for panel (D), and 10 μm for panel (E).

Additionally, we investigated the shaping of a phase-changing liquid droplet and film formation on the air–ferrofluidic interface. Structure formation on the air–liquid interface has been little studied besides forming ring-like or bowl-like structures using acoustic fields and drop evaporation.^31^ Due to the oscillations in the droplet caused by the acoustic fields, only certain shapes related to frequency modes can be formed. Here, we fix the shaped droplets through phase change using a solution of polystyrene mixed with toluene. Due to the high volatility of toluene, it evaporates within a few seconds and the solution then solidifies into a film on the air–ferrofluid interface, which could be later manipulated as a solid object. By actuating different coils during the solidification phase, we can control the shape of the fabricated film. Figure 5A-i and B-i shows two trials of forming X-shaped films at the air–ferrofluid interface using ∼1 μL of a 1.7 mg/mL polystyrene/toluene solution (Movie S5 part I). The solenoids were actuated with a ramp-up signal to 39 mT in 200 milliseconds and held at that level until the film solidifies. Figure 5A-ii and B-ii shows the solidified film at the interface. Figure 5C-i and C-ii shows the film formation of 50 mg/mL polystyrene/toluene solution (Movie S5 part II). Although the final shape is difficult to control precisely when the concentration is higher, the actuation of the solenoids still influences the final shape to elongate orthogonally to the actuated solenoids. When the concentration is lower, however, the final shape was more controllable. Figure 5D shows a zoomed-in micrograph of the formed film with ∼1 μL of 50 mg/mL polystyrene/toluene solution under an optical microscope. The film has a texture similar to crumpled plastic. The black particles visible on the film are iron nanoparticles from the ferrofluid. The scanning electron microscopy (SEM) image of the polystyrene film is shown in Figure 5E. The thickness of the film was approximately 450 nm, which contains a ∼0–20 wt % of iron content for the formed polystyrene films depending on the analyzed region (see Supporting Figure S6, Technical Note S2). Surface deformations of the formed films are observed during the solidification, which we attribute to the local stress of the film during evaporation.

## Conclusions

We reported a novel method for shaping liquid droplets on a programmable deformable air–ferrofluid interface both statically and dynamically. We have experimentally demonstrated that we can use multiple solenoids to deform a ferrofluidic interface to shape low-surface-tension droplets floating on the air–ferrofluid interface, e.g., hexadecane. We can shape the droplets in a variety of shapes, including controlling the aspect ratio of an elliptic-shaped droplet and creating quasi-static shapes, e.g., ellipses, half-moons, triangle-like shapes, and quadrilateral-like shapes. Depending on the spread of the oil droplet, both concave- and convex-shaped triangle-like shapes and quadrilateral-like shapes can be formed. For high-spreading liquids such as silicon oil, we can also stir the oil droplet. A frequency of 10 RPS or high is needed to stir the oil droplet to form spirals, whereas the viscosity of the oil will also impact the shape of the formed spirals being either more droplet-dominating or ribbon-dominating. We have also demonstrated that phase-changing liquid materials can also be shaped and solidified to form preprogrammed shapes at the air–ferrofluid interface, and the resulting centimeter-sized solid shapes have a thickness of about half a micron.

To the best of our knowledge, this is the first work on shaping liquid droplets at an air–liquid interface. The number and the placement of the solenoids can potentially be configured differently for more sophisticated droplet manipulation, e.g., splitting and merging. The proposed method could be exploited for forming films of various morphologies at the air–ferrofluid interface. The proposed method could open up new possibilities for tissue engineering and biological experiments when a biocompatible ferrofluid is used^32^ or potentially used as an active environment for mammalian cells, which adjust their internal structure, gene expression, and phenotype in response to their mechanical environment.^33^

## Experimental Section

### Experimental Setup

The experimental setup consists of eight electromagnetic solenoids (see Supporting Figure S1), adapted from our previous work for manipulating solid particles.^24^ The workspace from the top view is a circle with a diameter of up to 8 mm. We used continuous waveforms in this work by employing eight current controllers (ESCON 50/5, Maxon Group, Switzerland) to drive the solenoids. Using continuous waveforms, we can actuate the solenoids in a ramp to improve the stability of the ferrofluidic manipulation as it minimizes vibrations on the fluid surface compared to step actuation. The waveforms were generated using an analog output device (PCIe-6738, National Instruments). A camera (Point Gray GS3-U3-41C6C-C, FLIR Systems Inc., Canada) is used for visual feedback of the top view of the workspace. To illuminate the workspace, we use a 50:50 beam splitter (CCM1-BS013, Thorlabs Inc.) and an LED (MNWHL4, Thorlabs Inc.) coupled with a collimation lens. The image and data recording were performed using an in-house developed C++-based software. The color videos were recorded at a 10 Hz frequency. For the dynamic tests, we used a video recording rate of 50 Hz in grayscale. Due to the cyclic variations in illumination caused by the deformation of the ferrofluid, an elliptical object tracker was used to estimate droplet rotation.^34^ Further information on the experimental setup is available in Technical Note S3.

### Materials

We used a commercial water-based ferrofluid (EMG 408, Ferrotec) and diluted the ferrofluid for our liquid manipulation experiments. The nanoparticles in the ferrofluid have a nominal diameter of 10 nm. The EMG 408 ferrofluid was diluted at a ratio of 1:7 with Milli-Q Ultrapure water by volume to achieve the desired saturation magnetization for our liquid manipulation experiments (approximately 0.82 mT).

### Surface and Interfacial Tension Measurement

The surface tension of the liquids was measured using the pendant drop method using an optical tensiometer (Theta Flex, Biolin Scientific). For the interfacial tension measurement, a 11 × 11 × 45 mm quartz cuvette was filled with the manipulated oil, a pipette tip filled with the ferrofluid was immersed in the oil, and a droplet of the ferrofluid was dispensed to hang on the tip. The measurements were taken for 10 s and repeated three times, and the average values are shown in Supporting Section S5.

### Rheological Measurement

Rheological measurements were performed using a rheometer (Physica MCR 302, Anton Paar). A parallel plate geometry (50 mm radius) with 1.00 mm gap was used for measuring the silicon oils. Due to low viscosity and high volatility, a double gap geometry (DG26.7/T200/Stainless steel, Anton Paar) was used for measuring hexadecane and ferrofluid. Viscosity was measured with a shear rate sweep by increasing the shear rate from 0.1 to 100 (1/s) at 20 °C. (See Supporting Figure S7, Table S1). Measurements with torque values below 1 μN·m were removed due to high uncertainty.

### Filling of Ferromagnetic Liquid

The volume of the ferrofluid dish used for the experiments is 1.36 mL. Initially, the dish is overfilled with ferrofluid using a glass pipette. Then, the air–ferrofluid interface is flattened by removing the excess fluid. The ferrofluid was filled before each experiment to maintain a similar experimental condition. The ferrofluid was also refilled before the convex shaping experiments to compensate for the evaporation after the relatively long concave shaping experiments.

### Manipulated Liquids

In the manipulation experiments, we used *n*-hexadecane (H6703, Sigma-Aldrich) and silicon oil with 10 and 1000 cSt viscosities, respectively. Apart from oils, we also manipulated polystyrene/toluene solutions. The chosen liquids are immiscible with the ferrofluid and are less dense than the ferrofluid; therefore, they float at the air–ferrofluid interface.

### Centering of the Droplet and Solenoid Demagnetization

To improve the repeatability of the shaping experiments, the hexadecane droplet at the air–ferrofluid droplet is centered by the actuation of all solenoids with 35 mT field strength at the tip of each solenoid for five seconds (see Supporting Figure S3). Then, all solenoids are demagnetized before actuation of the desired solenoids for a shaping experiment with an exponentially decaying sinusoidal current signal given by *I* = *A*e^–*kt*^ Sin (ω*t*), where amplitude *A* = 2 A, rate of decay *k* = 2.5, and the sine frequency ω = 50 rad s^–1^ (see Supporting Figure S4).

### Elemental Distribution Analysis

The elemental distribution of the formed polystyrene films at the air–ferrofluid interface was analyzed using a scanning electron microscope (SIGMA VP, ZEISS) and energy-dispersive X-ray spectroscopy. The polystyrene films were transferred from the air–ferrofluid interface to a glass slide and dried in a desiccator overnight. Then, the films were sputter-coated with 10 nm of carbon prior to the elemental distribution analysis.

## Supporting Information Available

Experimental setup (Figure S1), magnetic flux density at the tip of each solenoid (Figure S2), demagnetization of the solenoids (Figure S3), centering of the droplets (Figure S4), sinusoidal actuation waveform for the rotation of oil droplet (Figure S5), energy-dispersive spectroscopy of the formed polystyrene films (Figure S6), rheological measurements of the liquids (Figure S7), properties of the manipulated liquids (Table S1), interfacial tensions between the liquids (Table S2), spreading coefficients of the manipulated liquids (Technical Note S1), energy-dispersive spectroscopy of the formed polystyrene films (Technical Note S2) (PDF)
Squeezing a hexadecane droplet at the air–ferrofluid interface using two opposite solenoid actuations (MP4)
Shaping of a hexadecane droplet at the air–ferrofluid interface using multiple solenoid actuations (MP4)
Rotation of a hexadecane droplet at the air–ferrofluid interface (MP4)
Stirring of liquid dispersed at the air–ferrofluid interface (MP4)
Formation of polystyrene films at the air–ferrofluid interface (MP4)
