# Supplementary material for: Shaping Liquid Droplets on an Active Air–Ferrofluid Interface
Source: Langmuir. 2023 May 24;39(22):7623–31. doi: 10.1021/acs.langmuir.3c00298 (PMC10249407; doi:10.1021/acs.langmuir.3c00298)
Supplement: Supplementary file 1 — la3c00298_si_001.pdf [file la3c00298_si_001.pdf]

## *Supporting Information*

# Shaping liquid droplets on an active air-ferrofluid interface

*P. A. Diluka Harischandra[1], Teemu Välisalmi[2], Zoran M. Cenev[3], Markus B. Linder[2], Quan Zhou[1,*]*

[1]Department of Electrical Engineering and Automation, School of Electrical Engineering, Aalto University, 02150 Espoo, Finland

[2]Department of Bioproducts and Biosystems, School of Chemical Engineering, Aalto University, FI-00076 Aalto, Finland

[3]Department of Applied Physics, School of Science, Aalto University, 02150 Espoo, Finland

E-mail: diluka.harischandra@aalto.fi; teemu.valisalmi@aalto.fi; zoran.cenev@aalto.fi; markus.linder@aalto.fi; quan.zhou@aalto.fi*

## Table of contents

Figure S1: Experimental setup

Figure S2: Magnetic flux density at the tip of each solenoid

Figure S3: Demagnetization of the solenoids

Figure S4: Centering of the droplets

Figure S5: Sinusoidal actuation waveform for rotation of oil droplet

Figure S6: Energy dispersive spectroscopy of the formed polystyrene films

Figure S7: Rheological measurements of the liquids

Table S1: Properties of the manipulated liquids

Table S2: Interfacial tensions between liquids

Technical note S1: Spreading coefficients of the manipulated liquids

Technical note S2: Energy dispersive spectroscopy (EDS) of the formed polystyrene films

Technical note S3: Experimental setup

Other supporting information: Movie S1 to S5 (5 movies)

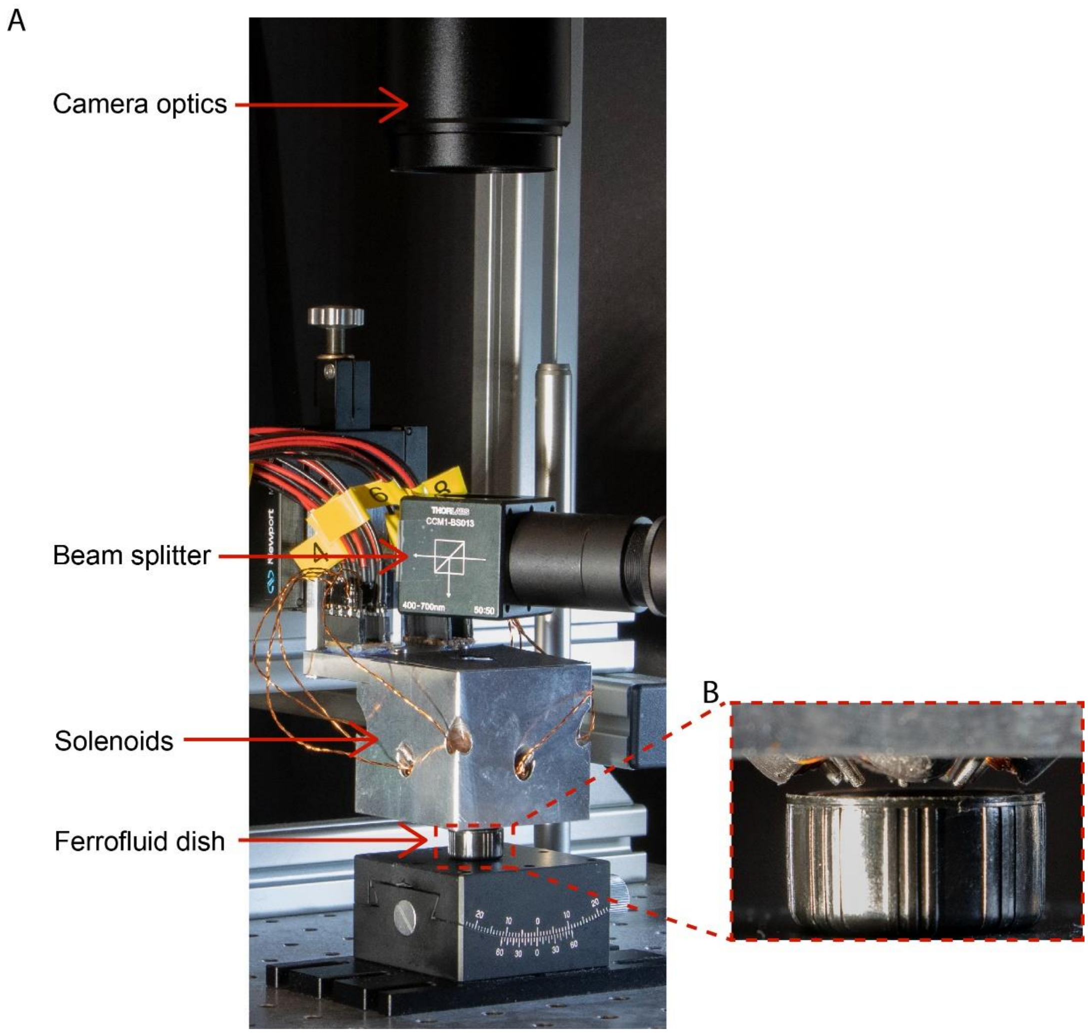


**Figure S1.** A) Experimental setup B) Close up view of the solenoid tips and the ferrofluid dish.

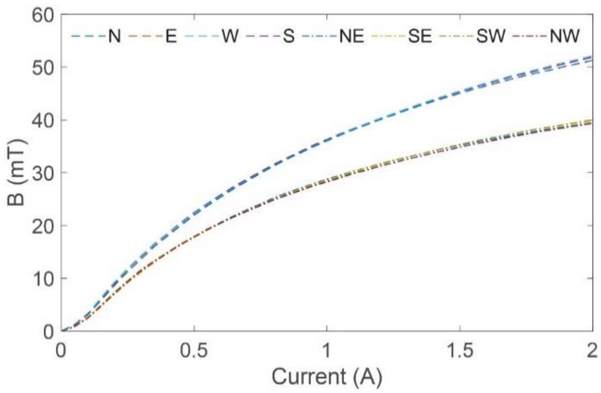


**Figure S2.** Magnetic flux density at the tip of each solenoid.

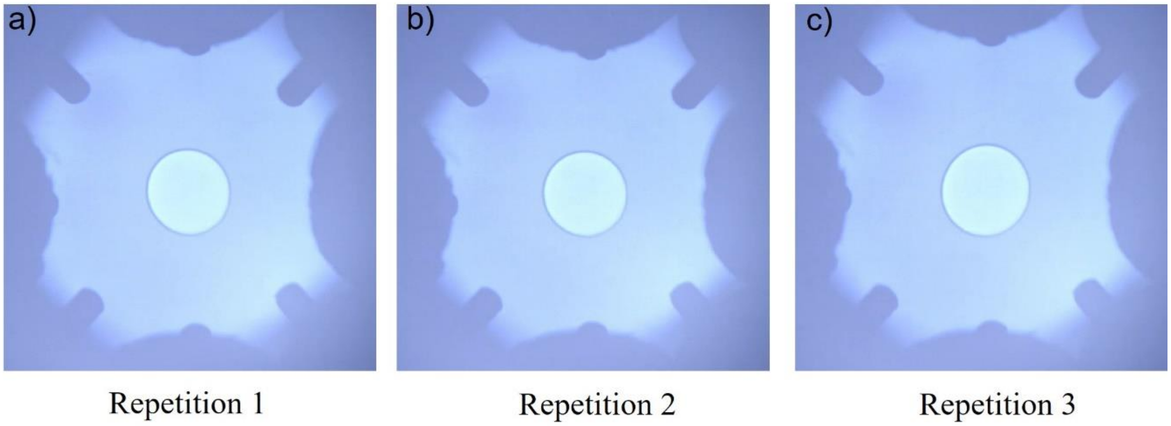


**Figure S3**. Centering of the droplet

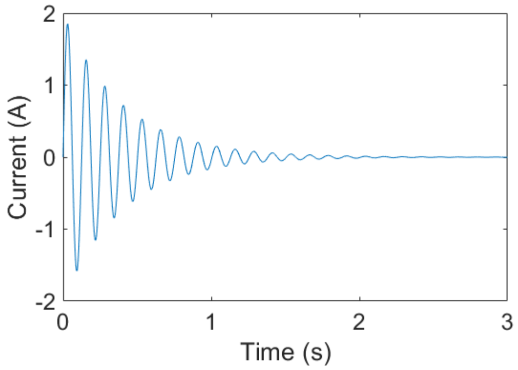


**Figure S4.** Current waveform for demagnetization of the solenoids

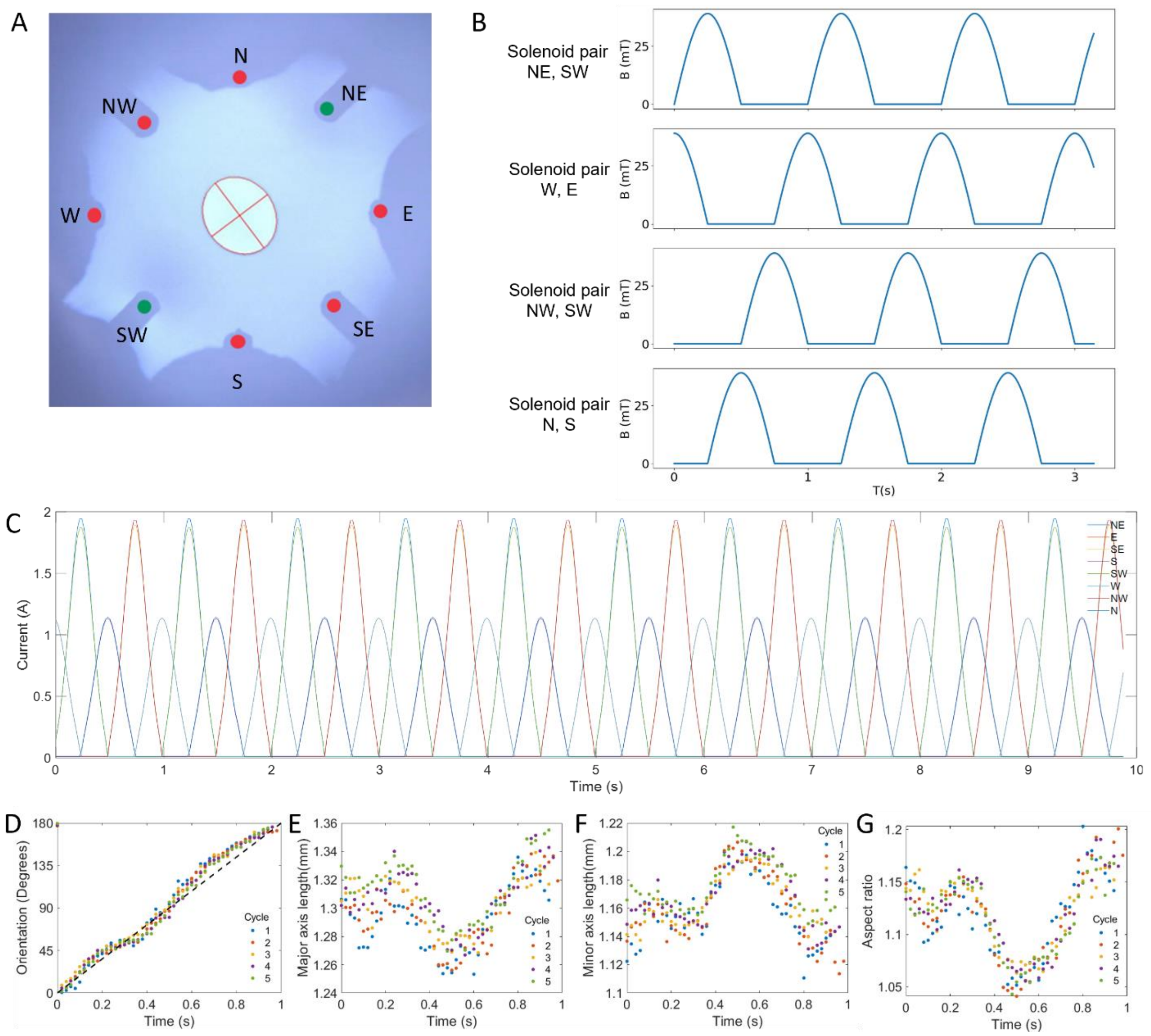


**Figure S5.** Sinusoidal actuation waveform for rotation of oil droplet. (A) Shape of the droplet when two opposite solenoids are actuated simultaneously. (B) Waveforms used for droplet rotation at the air-ferrofluid interface. (C) Generated current signals. Raw data for the (D) angle response of the rotating droplet. (E) Major axis length of the droplet. (F) Minor axis length of the droplet. (G) The aspect ratio of the droplet.

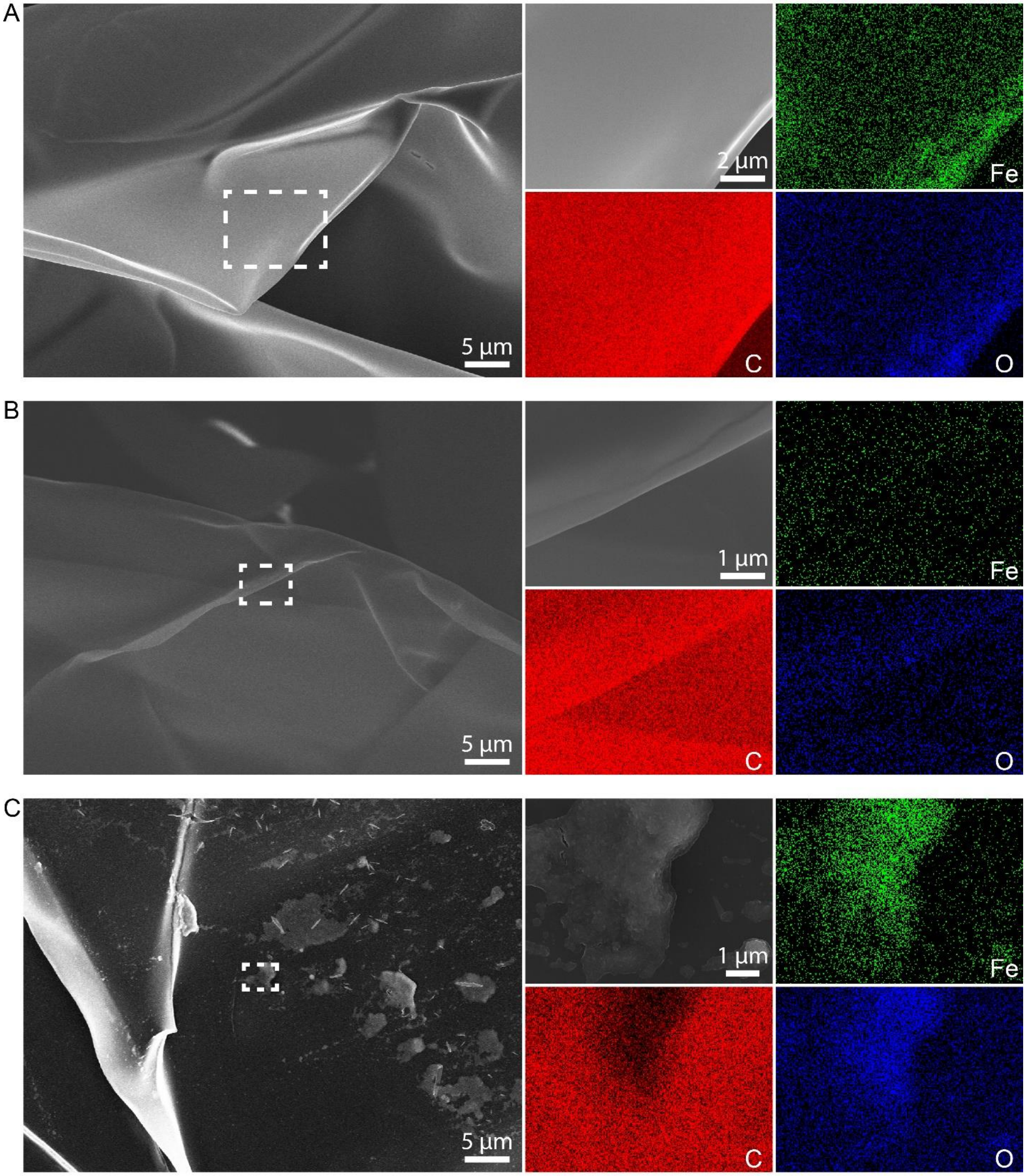


**Figure S6.** (A)-(C) Energy dispersive spectroscopy in different regions of a polystyrene film.

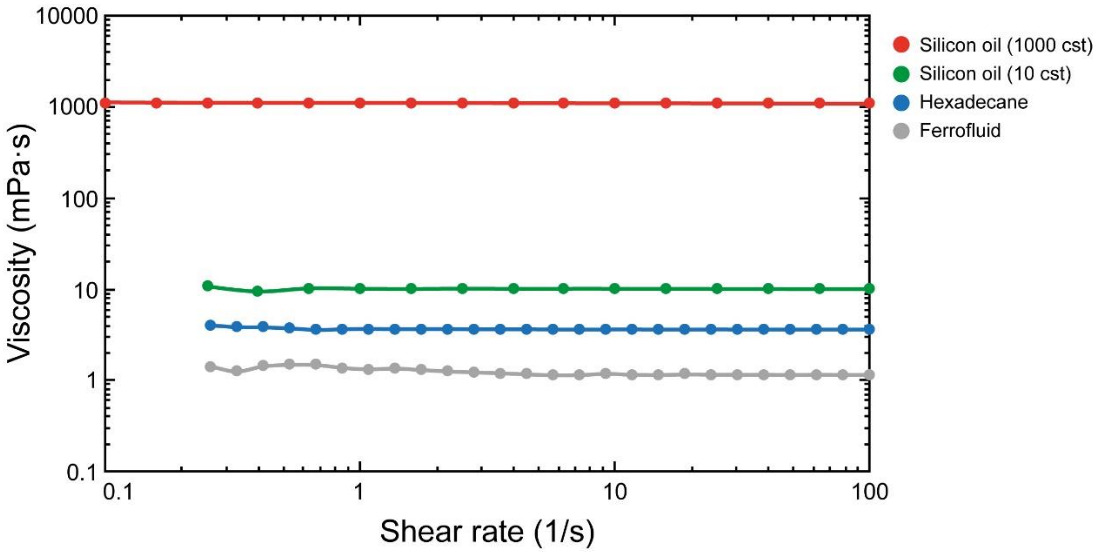


**Figure S7.** Rheological measurements of the liquids. All the materials showed Newtonian behavior.

**Table S1**. Properties of the manipulation liquids

| Liquid | Surface tension (mN $m^{-1}$) | Density ($gcm^{-3}$) | Viscosity (mPa·s) |
|---|---|---|---|
| Ferrofluid | 70.88 | 0.99123 | 3.558 |
| Hexadecane | 27.39 | 0.76851 | 1.138 |
| Silicon oil (10 cSt) | 20.1* | 0.93225 | 10.211 |
| Silicon oil (1000 cSt) | 21.2* | 0.96388 | 1106.025 |

*Values obtained from literature[1]

**Table S2.** Interfacial tensions between liquids

| Interface | Interfacial tension (mN $m^{-1}$) |
|---|---|
| Ferrofluid - Hexadecane | 41.85 |
| Ferrofluid - Silicon Oil (10 cSt) | 34.07 |

**Technical note S1**

The spreading coefficients for the manipulated liquids can be described with $S = \gamma_{AF} - \gamma_{OA} - \gamma_{OF}$ , where $\gamma_{AF}$, $\gamma_{OA}$ represent the surface tensions of the ferrofluid and oil droplet and $\gamma_{OF}$ represent the interfacial tension of the oil droplet and the ferrofluid. Hexadecane and silicon oil with a viscosity of 10 cSt have spreading coefficients of 1.64 and 16.71, respectively.

**Technical note S2**

Energy dispersive spectroscopy (EDS) data suggests that iron content was generally within several wt%, but there were local differences. Edges of the polystyrene film shown in Figure S6A contained higher concentration of iron (1.4 wt%), while some areas were found to contain no iron at all (Figure S6B). In some cases, the iron particles formed aggregates on the surface of the film increasing the local iron content substantially (19.4 wt%) (see Figure S6C). Other major elements were carbon and oxygen, which likely originate from polystyrene and $Fe_2O_3$ nanoparticles in the ferrofluid, respectively. However, it should be

noted that quantitative analysis of light elements with EDS is prone to error and thus the wt% values are approximations.

### Technical note S3

The solenoid coils are comprised of 300-400 turns of SWG 27 copper wire in six layers wrapped around a martensitic steel core with a diameter of 1 mm. To achieve a circular arrangement of the solenoids and due to geometric considerations, four of the solenoids have shorter tips and the other four have longer tips. All solenoids are placed about 1 mm above the air-ferrofluid surface. The analog output sample rate was set to 1 kHz for generating low-frequency signals ($f < 100$Hz) and 10 kHz for generating high-frequency signals ($f > 100$Hz). The current controllers are powered with 12 V fixed voltage using a low noise power supply (E36233A, Keysight, USA). For calibration purposes, the magnetic flux density at the tip of each solenoid was measured using a hall effect sensor (SS495A1, Honeywell, USA). The resultant magnetic flux density for actuation currents up to 2 A is shown in the supplementary Figure S2. The B-I relations of individual solenoids were fitted to fifth-degree polynomials to generate currents needed to keep a consistent field strength at the tip of each solenoid. During simultaneous actuation of short and long solenoids for quasi-static shaping of droplets, we used 25 mT magnetic flux density at the solenoid tips. The magnetic flux density for shaping droplets was kept at 25 mT to avoid pushing the droplet outside the workspace during the shaping process. We used a 39 mT peak field strength at the solenid tips for droplet rotation and stirring experiments (see Supplementary Figure S5).

### Supplementary Movies

S1: Squeezing a hexadecane droplet at the air-ferrofluid interface using two opposite solenoid actuations.

S2: Shaping of a hexadecane droplet at the air-ferrofluid interface using multiple solenoid actuations.

S3: Rotation of a hexadecane droplet at the air-ferrofluid interface.

S4: Stirring of liquid dispersed at the air-ferrofluid interface.

S5: Formation of polystyrene films at the air-ferrofluid interface.

### References

(1) Milionis, A.; Antonini, C.; Jung, S.; Nelson, A.; Schutzius, T. M.; Poulikakos, D. Contactless Transport and Mixing of Liquids on Self-Sustained Sublimating Coatings. *Langmuir* **2017**, *33* (8), 1799–1809. https://doi.org/10.1021/acs.langmuir.6b04377.
